# Adenoma and carcinoma in the anal transitional zone following hand-sewn versus stapled ileal pouch-anal anastomosis in familial adenomatous polyposis

**DOI:** 10.1007/s10689-025-00498-0

**Published:** 2025-10-23

**Authors:** Jun Yong Han, Min Jung Kim, Sang Hui Moon, Young Jin Kim, Hyun Tae Lim, Jesung Park, Jae Hyun Park, Hyo Jun Kim, Ji Won Park, Seung-Bum Ryoo, Kyu Joo Park, Seung-Yong Jeong

**Affiliations:** 1 Department of Surgery, Seoul National University College of Medicine, 101, Daehak-ro Jongno-gu, Seoul, 03080 Republic of Korea; 2 Colorectal Cancer Center, Seoul National University Cancer Hospital, Seoul, Republic of Korea; 3 Cancer Research Institute, Seoul National University, Seoul, Republic of Korea

**Keywords:** Familial adenomatous polyposis, Ileal pouch-anal anastomosis, Anal transitional zone, Adenoma recurrence

## Abstract

Restorative proctocolectomy (RPC) with ileal pouch-anal anastomosis (IPAA) is the standard surgical treatment for familial adenomatous polyposis (FAP) in our institution. Stapled and hand-sewn IPAA differ in the amount of retained rectal mucosa, influencing both adenoma risk and functional outcomes. In this retrospective cohort study, we compared oncological and functional outcomes between the two techniques. Consecutive patients with FAP who underwent RPC with IPAA at Seoul National University Hospital between 1994 and 2022 were included. The ATZ adenoma and cancer occurrence, operative time, and anorectal functional outcomes were compared between hand-sewn and stapled anastomosis groups. The cumulative incidence risk of adenoma recurrence was compared using the log-rank test. 89 patients were analyzed: 32 underwent hand-sewn and 57 underwent stapled IPAA. Median follow-up was 100 months in the stapled group and 79 months in the hand-sewn group. Stapled IPAA was associated with shorter operative time (220.6 vs. 332.9 min, *p* < 0.001), lower diverting ileostomy rate (78.9% vs. 96.9%, *p* = 0.027), and lower Wexner incontinence scores (1.54 vs. 3.43, *p* = 0.009). Adenomas or carcinomas at the ATZ were identified in 29.2% of patients, with a higher 5-year adenoma recurrence in the stapled group (20.1% vs. 3.2%, *p* = 0.02). Most adenomas were successfully managed endoscopically, with no cancer progression in the stapled group. Two cancers occurred in the hand-sewn group during long-term follow-up. Stapled IPAA offers advantages in operative efficiency and anorectal function, with higher adenoma recurrence manageable under surveillance. These findings suggest stapled IPAA may be a reasonable surgical option for FAP when coupled with diligent follow-up.

## Introduction

Familial adenomatous polyposis (FAP) is the autosomal dominant hereditary polyposis syndrome caused by germline pathogenic or likely pathogenic variants in the *APC* gene [1]. Patients with FAP inherit a mutated copy of the *APC* gene, leading to the early development of numerous colorectal adenomas. These adenomas can eventually progress to cancer through the acquisition of additional somatic mutations in oncogenes. Although surgical management of familial adenomatous polyposis (FAP) varies worldwide, two primary approaches are commonly performed: ileorectal anastomosis (IRA) and restorative proctocolectomy (RPC) with ileal pouch-anal anastomosis (IPAA). In our institution, however, RPC with IPAA has been the standard surgical treatment for FAP to achieve complete removal of colorectal mucosa [2].

There are two primary techniques of IPAA: stapled and hand-sewn. Since the initial reports by Parks and Nicholls in 1978, all IPAAs were performed using the hand-sewn method until the 1980’s when the stapled technique was introduced with the invention of the circular stapler [3, 4]. The key difference between these two techniques lies in the length of the remnant rectal mucosa at the anal transitional zone (ATZ). The stapled method employs a circular stapling device to connect the ileal pouch to the anal canal. This technique requires a rectal stump for stump deployment, which inevitably leaves some rectal mucosa near the ATZ [5]. In contrast, hand-sewn method typically involves a trans-anal mucosectomy of the remnant rectum, followed by a manually sutured anastomosis [6].

The potential impact of the remnant mucosa on adenoma recurrence and surgical outcomes remains controversial [7]. The choice between stapled and hand-sewn technique is often based on several clinical factors, including the surgeon’s experience, the condition of the rectal cuff, the severity of distal rectal polyp burden, and the patient’s anatomical considerations. While some studies suggest that the remnant rectal stump in stapled IPAA is associated with a higher rate of adenoma recurrence in the anorectal segment, the long-term risk of these adenomas progressing to clinically significant cancer has not clearly investigated [8, 9]. Additionally, extensive mucosectomy in the hand-sewn method may exacerbate postoperative anal incontinence [10]. Therefore, balancing between surgical morbidity and risk of cancer at ATZ warrants further evaluation.

This study was designed to compare adenoma recurrence in the anorectal segment, surgical outcomes, and functional results between stapled and hand-sewn IPAA techniques. In addition to assessing short-term recurrence rates, we have tracked the life-long prognosis and outcomes of patients with anorectal segment adenoma.

## Methods

### Study design

This was a retrospective cohort study conducted at the tertiary referral cancer center. Data were extracted from a prospectively maintained database in the Department of Surgery, Seoul National University Hospital (SNUH), which includes patients with colorectal cancer and hereditary colorectal syndromes. This study complies with the Declaration of Helsinki and was conducted following ethics committee approval. This study was approved by the Institutional Review Board (IRB) of Seoul National University Hospital (IRB no. 2410-071-1576).

### Patients

Patients who underwent RPC with IPAA due to FAP from 1994 to 2022 were selected from the SNUH database. Only patients who have received endoscopic surveillance were included. Patients were excluded if they lack information about the type of anastomosis or endoscopic surveillance. Additionally, patients who underwent ileo-rectal anastomosis or end ileostomy without IPAA were excluded.

### Variables and outcomes

Collected variables include baseline demographics such as age, sex, body mass index, anastomosis type, family history of FAP, presence of colorectal cancer, and polyp density in the colon. Sigmoidoscopic records, images, and pathology from sigmoidoscopic biopsies were reviewed to assess the recurrence of adenomas or cancers in the ATZ. The primary end points included the adenoma recurrence rate and cumulative risk of adenoma recurrence in the ATZ. Secondary end points included the occurrence of cancer in the ATZ, treatment methods for recurrent adenomas in the ATZ, the operative time for RPC with IPAA, the diverting ileostomy rate, and the severity of fecal incontinence during follow-up.

### Definitions

An adenoma in the ATZ was defined as any adenoma found from the anus to the anastomosis site during endoscopic surveillance after RPC with IPAA [11]. To minimize the possibility of misclassifying residual adenomas, all adenomas detected during follow-up endoscopy were initially regarded as recurrence. When adenomas were identified at the first follow-up endoscopy, detailed comparison with preoperative rectal endoscopic findings was performed to differentiate residual from newly developed lesions. Adenomas detected only at terminal ileum or ileal J-pouch (n = 3) were excluded from the definition of anorectal segment adenoma recurrence. Polyps were classified according to Paris classification, and biopsies were performed on adenomatous-appearing polyps [12]. Benign findings, such as cryptitis, ileitis or lymphoid follicles on pathological examination, were not considered adenoma recurrence.

Operative time was defined as the interval time from the start of surgery to its completion, as recorded in anesthesia and operative logs. Postoperative complications such as ileus, recto-vesical fistula, and anastomosis disruption, which could impact on postoperative bowel function, were documented.

The severity of fecal incontinence was assessed using the Wexner score. For this study, the scores were collected cross-sectionally at the time of data analysis through structured telephone interviews with all available patients, regardless of the interval after surgery. The median interval between surgery and Wexner score assessment was 76 months (range, 26–204 months). [13] The Wexner score is the sum of 5 categories, including the frequency of fecal incontinence for solid stool, liquid stool, and gas, the use of pads, and lifestyle alterations. Each category is scored from 0 to 5, with 0 representing “never” and 5 presenting “always”. A Wexner scores ≥ 1 was considered to be symptomatic (1–4: mild incontinence, 5–8: moderate incontinence, 9–20: severe incontinence).

### Surgical procedure and endoscopic surveillance

RPC with IPAA was performed by seven experienced surgeons using open, laparoscopic, or robotic total mesorectal excision. Following dissection of the entire colon and rectum, the right, middle, and left colic arteries, as well as the sigmoidal artery, were ligated, and a total proctocolectomy was completed. An ileal J-pouch was constructed, and the IPAA was performed. In some cases, RPC was not accompanied by IPAA, such as patients who had undergone a previous total colectomy or Hartmann’s procedure.

Stapled anastomoses between the anus and ileal J-pouch were performed using a circular stapling device. Hand-sewn anastomoses were completed following trans-anal mucosectomy and manual interrupted sutures.

Endoscopic surveillance was routinely conducted after RPC with IPAA using flexible sigmoidoscopy. In our institution, pouchoscopy or sigmoidoscopy was typically performed within 6–12 months after surgery, and subsequently every 1–2 years depending on the presence of adenomas or other abnormal findings. If adenomatous polyps were detected, they were removed through endoscopic polypectomy or endoscopic mucosal resection during procedure. Polyps that could not be removed endoscopically due to location, size, or previous interventions were excised trans-anally under spinal or general anesthesia.

### Statistical analysis

Patients were categorized into two groups based on the type of anastomosis: stapled and hand-sewn. Pearson’s chi-square test, Fisher’s exact test, or the Mann–Whitney U test was used to compare categorical and continuous variables. The Kaplan–Meier method and log-rank test were employed to calculate the cumulative incidence of adenoma recurrence. A swimmer’s plot was used to visualize the timeline of adenoma recurrence. Statistical analyses were performed using IBM SPSS Statistics, version 26 (IBM Corp., Armonk, NY, USA). A *p*-value of < 0.05 was considered statistically significant.

## Results

### Clinical characteristics

**Fig. 1:**
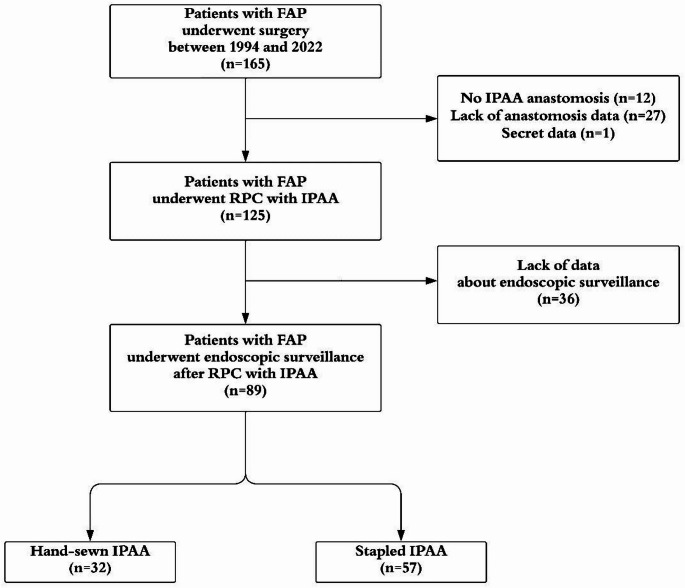
Flow diagram of patient selection. FAP, familial adenomatous polyposis; IPAA, ileal pouch-anal anastomosis; RPC, restorative proctocolectomy; SD, standard deviation; BMI, body mass index

Between 1994 and 2022, a total of 165 FAP patients underwent surgery, and 124 patients received RPC with IPAA (Fig. 1). Patients who underwent other types of anastomoses such as ileorectal anastomosis (n = 9), end ileostomy (n = 3), those with incomplete data on the type of anastomosis (n = 27), and one patient with privacy records were excluded. Endoscopic follow-up data were unavailable in 36 patients. Among them, 8 patients underwent surgery in the early years of the study, and their endoscopic procedure records could not be retrieved due to missing archived optical scan data. The remaining 28 patients were either lost to follow-up or continued their surveillance at local hospitals, where endoscopic records were not accessible. After excluding 36 patients, 89 patients were included in the study: 32 patients underwent hand-sewn IPAA, and 57 patients received stapled IPAA. Among the 32 patients who underwent hand-sewn IPAA, 9 (28.1%) were performed between 1994 and 2004, 8 (25.0%) between 2005 and 2015, and 15 (46.9%) between 2016 and 2022. In contrast, of the 57 patients who underwent stapled IPAA, only 2 (3.5%) were performed between 1994 and 2004, whereas the majority were performed after 2005, with 30 cases (52.6%) between 2005 and 2015 and 25 cases (43.9%) between 2016 and 2022. These data indicate that hand-sewn IPAA was the predominant technique in the earlier period, but stapled IPAA gradually became the dominant approach over time. Nevertheless, some surgeons still preferred hand-sewn IPAA in selected cases to ensure complete removal of the rectal mucosa.

Table 1 presents the baseline characteristics of the included FAP patients. Of the 28 years of cumulative data, approximately 69% of all surgeries were performed within the last 14 years. The mean age at the time of surgery was 31.5 years, and 58% of patients had a known family history of FAP. Four cases involved previous colorectal surgery, including Hartmann’s procedure and low anterior resection due to colorectal cancer before the diagnosis of FAP.

**Table 1:** Clinical characteristics of patients diagnosed with FAP

Values	Total (n = 89)	Hand-sewn IPAA (n = 32)	Stapled IPAA (n = 57)	*p*-value
*Sex, n (%)*				
Male	49 (55.1)	17 (53.1)	32 (56.1)	0.784
Female	40 (44.9)	15 (46.9)	25 (43.9)	
Age at the operation, yr, mean ± SD	31.5 ± 12.9	33.0 ± 11.8	30.7 ± 13.6	0.420
BMI, kg/m^2^, mean ± SD	22.4 ± 4.0	22.4 ± 5.0	22.5 ± 3.3	0.950
Comorbidity, n (%)	19 (21.3)	7 (21.9)	12 (21.1)	0.928
Family history of FAP, n (%)	51 (58)	17 (54.8)	34 (59.6)	0.662
*Year of operation, n (%)*				
Before 2008	28 (31.5)	11 (34.4)	17 (29.8)	0.657
After 2009	61 (68.5)	21 (65.6)	40 (70.2)	
Previous colorectal surgery (%)	4 (4.5)	2 (6.3)	2 (3.5)	0.617

FAP, familial adenomatous polyposis; IPAA, ileal pouch-anal anastomosis; SD, standard deviation; BMI, body mass index

### Peri-operative characteristics

Table 2 compares the peri-operative characteristics between the two groups. Ileostomy diversion was performed in 96.9% of patients with hand-sewn IPAA, compared to 78.9% of patients with stapled IPAA (*p* = 0.027). The mean operative time was significantly shorter in the stapled IPAA group compared to the hand-sewn group (220.6 ± 71.9 vs. 332.9 ± 102.6 min, *p* < 0.001). There were no significant differences between the groups in terms of postoperative complications, polyp density, or the presence of colorectal cancer at the time of surgery.

**Table 2:** Operative outcomes of patients with FAP who underwent RPC with IPAA using the hand-sewn vs. stapled techniques

Values		Total (n = 89)	Hand-sewn IPAA (n = 32)	Stapled IPAA (n = 57)	*p*-value
Operative approach, n (%)	Open	64 (71.9)	19 (59.4)	45 (h78.9)	0.078
	Laparoscopic	24 (27.0)	12 (37.5)	12 (21.1)	
	Robot-assisted	1 (1.1)	1 (3.1)	0 (0.0)	
Ileostomy diversion, n (%)		76 (85.4)	31 (96.9)	45 (78.9)	0.027
Mean operation time, min (SD)		260.2 (99.4)	332.9 (102.6)	220.6 (71.9)	< 0.001
Postoperative complication, n (%)	Ileus	8 (9)	2 (6.3)	6 (10.5)	0.226
	Fistula	1 (1.1)	1 (3.1)	0 (0.0)	
	Anastomosis disruption	1 (1.1)	1 (3.1)	0 (0.0)	
Colon polyp density at the time of RPC with IPAA, n (%)	< 100	16 (18.0)	6 (18.8)	10 (17.5)	0.713
	100 ≤ n < 1000	58 (65.2)	22 (68.8)	36 (63.2)	
	≥ 1000	15 (16.9)	4 (12.5)	11 (19.3)	
Colorectal cancer at the time of RPC with IPAA	No cancer	50 (56.2)	19 (59.4)	31 (54.4)	0.120
	Stage I	21 (23.6)	4 (12.5)	17 (29.8)	
	Stage II–IV	18 (20.2)	9 (28.1)	9 (15.8)	
Follow-up duration, months (range)		92 (2–355)	79 (3–352)	100 (2–206)	0.178
Time to first endoscopic surveillance, months (range)		21 (1–219)	7 (1–219)	23 (1–203)	0.931
Adenoma recurrence, n (%)		26 (29.2)	6 (18.8)	20 (35.1)	0.104

FAP, familial adenomatous polyposis; RPC, restorative proctocolectomy; IPAA, ileal pouch-anal anastomosis; SD, standard deviation

### Adenoma recurrence at the anal transitional zone (ATZ)

The median total follow-up was 92 months (range, 2–355 months), with 79 months (range, 3–352) for the hand-sewn group and 100 months (range, 2–206) for the stapled group (*p* = 0.178). The median time to first endoscopic surveillance was 21 months [7 months (range, 1–219) in the hand-sewn group vs. 23 months (range, 1–203) in the stapled group, *p* = 0.178].

**Fig. 2:**
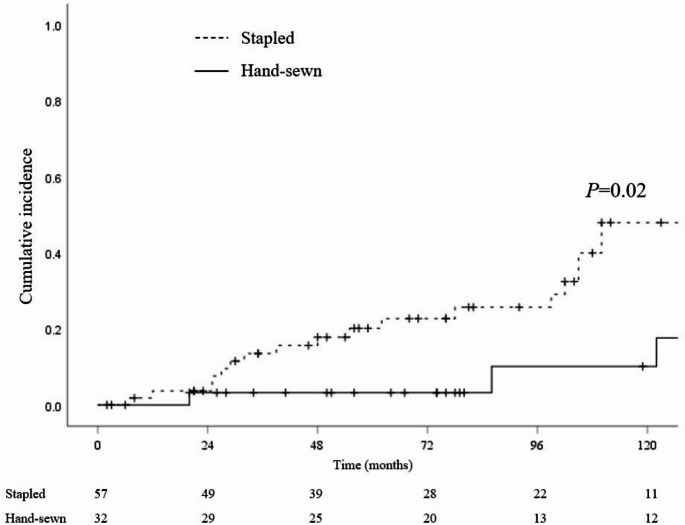
Cumulative risk of anorectal segment adenoma recurrence

During follow-up period, the recurrence rate of adenoma at the ATZ was 18.8% (6 of 32 patients) in the hand-sewn group and 35.1% (20 of 57 patients) in the stapled group (*p* = 0.104; Table 2). Cumulative adenoma recurrence rates demonstrated a higher and earlier incidence of adenoma and carcinoma recurrence in the stapled group (5-year adenoma recurrence; 3.2% in the hand-sewn group vs. 20.1% in the stapled group, *p* = 0.02; Fig. 2).

### Long-term timeline of adenoma recurrence cases

**Fig. 3:**
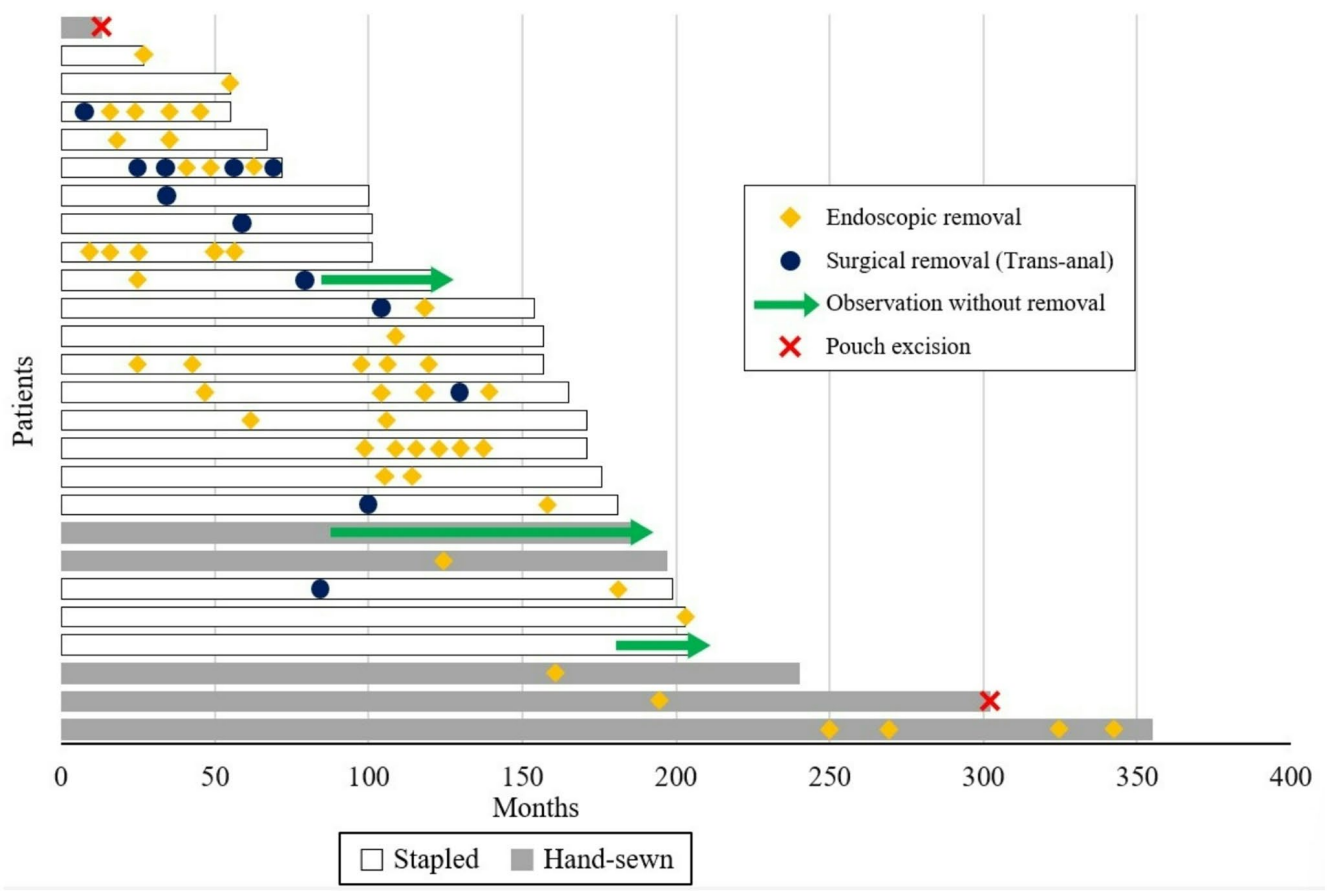
Swimmer’s plot of anorectal segment adenoma recurrence cases

To evaluate oncological outcomes and prognosis, the timeline of adenoma and carcinoma recurrence was tracked for 26 patients (Fig. 3). Adenoma recurrence was identified at a median of 84 months after surgery (range, 7–251 months). In our cohort, all cases with adenomas detected at the first surveillance endoscopy occurred more than one year after surgery, supporting the interpretation that they were de novo lesions rather than residual disease. Among these 26 patients, 21 patients achieved adenoma-free status after endoscopic or surgical adenoma removal. Seven patients required only a single procedure for successful polyp clearance, while 14 underwent repeated endoscopic or surgical removal of recurrent adenomas (1 in the hand-sewn group, 13 in the stapled group).

Only two cases from the hand-sewn group required pouch excision. One patient experienced early recurrence of the primary cancer in lymph nodes, while the other developed rectal cancer 25 years later after being lost to follow-up.

### Functional outcomes

**Fig. 4:**
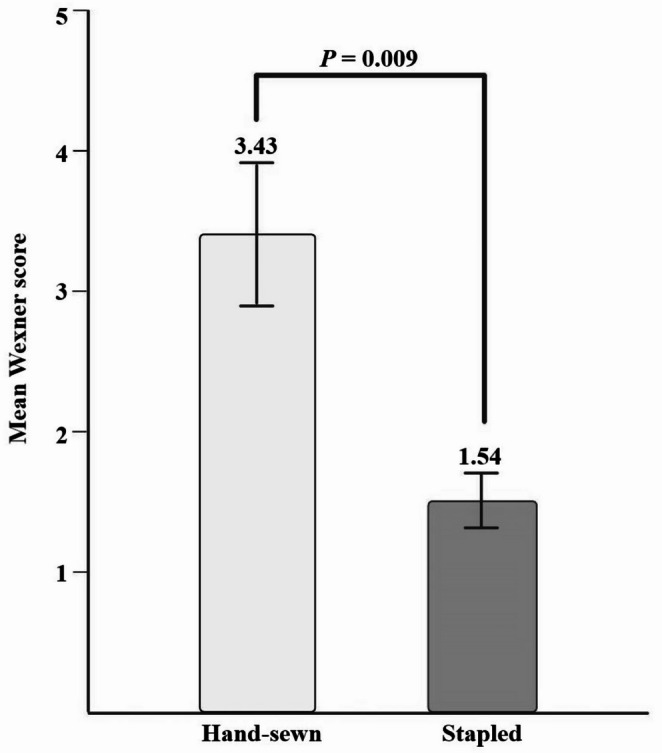
Average Wexner score

Of the 89 patients, 49 (14 in the hand-sewn group and 35 in the stapled group) participated in the telephone survey assessing anal incontinence. Thirty-one patients did not respond, and six patients who did not undergo ileostomy repair and three deceased patients were excluded. The average Wexner incontinence score was significantly higher in the hand-sewn group, including a higher degree of anal incontinence compared to the stapled group (3.43 vs. 1.54, respectively, *p* = 0.009; Fig. 4).

## Discussion

Our study demonstrated that the stapled technique for RPC with IPAA required less operative time, resulted in fewer diverting ileostomies, and caused less anal incontinence compared to the hand-sewn technique. Despite the higher rate of adenoma recurrence in the stapled group, with frequent surveillance, recurrent adenomas could be effectively cleared endoscopically or surgically. Notably, progression to cancer at the ATZ occurred only in the hand-sewn group, leading to the inference that close surveillance is more critical for cancer prevention than the choice of anastomosis technique.

The choice between stapled and hand-sewn IPAA was primarily determined by the surgeon’s preference rather than patient-related oncologic factors. As summarized in Table 2, there were no significant differences between the two groups with respect to colon polyp density or the presence of cancer at the time of surgery, indicating that rectal polyp burden was not a major determinant of anastomosis type in this cohort. Some surgeons favored the stapled technique for its time- and function-saving advantages, while others selected the hand-sewn method to perform trans-anal mucosectomy, aiming to eradicate all potential adenoma recurrence risks [14]. To identify the optimal surgical technique, this single center retrospective study analyzed and compared the long-term oncological and surgical outcomes between the two anastomosis groups.

By tracking the long-term course of patients with adenoma recurrence, we found that remnant rectal mucosa in the stapled method was a significant risk factor for repeated adenoma recurrence. During endoscopic evaluations, the rectal stump left for anastomosis in the stapled group could be easily identified upon macroscopic examination [15]. Of the 14 cases of recurrent adenoma following initial removal, 13 cases were in the stapled group. Seven patients underwent more than four adenoma removal procedures, 6 of whom had undergone stapled IPAA. In three cases, multiple polyps (> 10) were detected, but complete removal was not done due to their benign looking appearance. Biopsies confirmed these polyps as tubular adenoma, low grade, and they were monitored with short-term follow-up endoscopy. We also reviewed the types of procedures used for adenoma clearance. In nine cases, trans-anal polypectomy under spinal or general anesthesia was performed when adenomas were too large for endoscopic removal and located close to the anus—all of these cases were in the stapled group.

Our study confirmed that a small amount of rectal mucosa, known as remnant rectal mucosa, could be left behind and cause adenoma recurrence, with the amount varying depending on the anastomosis technique [16]. In 2015, Ganschow reported that residual rectal mucosa was found more frequently after stapled IPAA compared to the hand-sewn method based on microscopic reviews of 100 biopsy cases [17]. Von Roon’s 2011 study also demonstrated that adenoma recurrence in the anorectal segment was more frequent and occurred earlier in the stapled group during a 10-year follow-up of 120 cases [8]. They also categorized adenomas larger than 10 mm and cancer cases, noting that one adenocarcinoma occurred, while nine high-risk adenomas were successfully treated with local excision. Other studies also have similarly demonstrated a higher risk of adenoma recurrence with stapled methods compared to hand-sewn methods [9].

However, it is important to emphasize that, in our study, none of the patients with recurrent adenomas or multiple polyps developed adenocarcinoma in the stapled group. The median time to the first endoscopic surveillance was 20 months, indicating that patients typically received endoscopic follow-up within two years of surgery. More recent cases undergo endoscopy within six months, unless follow-up is lost or postoperative complications arise. This rigorous endoscopic surveillance appears to be preventing adenoma progression to cancer (Fig. 4). Previous studies have reported cases of cancer in residual rectal mucosa, but the proportion of adenomas progressing to cancer has remained very low [18–20].

In terms of surgical outcomes, stapled method was significantly faster (227.9 min vs. 324.5 min). The hand-sewn method prolongs surgery due to the mucosectomy and manual suturing required, and it is widely recognized that prolonged operative time can negatively affect patient prognosis [21–23]. Given the absence of a rectum in patients undergoing ileo-anal anastomosis, fecal incontinence is a major postoperative concern [24]. The Wexner score was used as a representative method of bowel function, and it was higher in the hand-sewn group, indicating worse anal function compared to the stapled group (3.43 vs. 1.54, *p* = 0.009).

To compare functional outcomes between the two groups, we also included complications related to bowel function such as ileus, recto-vesical fistula, and anastomosis disruption, while excluding non-bowel-related complications such as transfusion, pneumonia, and wound problem [25]. Although there was no significant difference in the overall complication rate between the two groups, major anastomotic complications occurred exclusively in the hand-sewn group. This aligns with many other studies suggesting that the stapled method offers better bowel function and time-saving advantages [26–28].

Several reports are consistent with our findings. In 2013, the Cleveland clinic compared the oncological and functional outcomes between two techniques, concluding that while the stapled method carried a higher risk of adenoma recurrence, it did not lead to a higher risk of carcinoma. In contrast, anastomosis stricture and fecal problem were more common in the hand-sewn method [10].

In our study, two patients from the hand-sewn group required pouch excision due to cancer development. One patient underwent abdominoperineal resection with pouch excision and lateral lymph node dissection after 20 months from RPC with IPAA due to lymph node metastasis detected on F-18 FDG PET-CT. This patient had sigmoid colon cancer at the time of initial RPC with IPAA. Metastatic adenocarcinoma from the sigmoid colon cancer was confirmed pathologically. This case is more appropriately considered a recurrence of the primary cancer rather than progression from adenoma. The other patient, diagnosed with sigmoid colon cancer 27 years ago, developed an adenoma at the dentate line 15 years postoperatively, which was removed endoscopically. After 10 years of follow-up loss, the patient presented with a large palpable anal mass, which was excised with ileal-pouch and confirmed as adenocarcinoma.

There are some limitations to our study. First, this was a retrospective study with a relatively small sample size. Our institution is a tertiary referral center, recognized as a large-volume cancer center in South Korea, performing approximately 1000 colorectal surgeries annually, including 500–600 operations for colorectal cancer. Considering that FAP accounts for approximately 1% of colorectal cancer cases, the number of cases in our cohort is not extremely small. On average, about four patients per year have undergone RPC with IPAA at our center; however, review of the most recent 3–5 years of surgical records shows that this number has increased to approximately seven cases annually. We anticipate that with continued systematic follow-up and case accumulation, a larger cohort will be available to further clarify the lifelong clinical course of FAP patients. Second, the Wexner score was obtained cross-sectionally, and because the time from the surgery varied between patients, it may not fully represent bowel function differences. A more systematic follow-up system is needed to collect functional outcomes of FAP patients over time. Third, we did not analyze the relationship between gene mutations and outcomes. For patients who underwent surgery more than 20 years ago, APC gene sequencing data were lacking. Future studies will explore the association between APC mutations and anorectal adenoma recurrence based on recent findings [29]. Fourth, the severity of polyp burden in the distal rectum might have influenced the choice of anastomosis technique. However, due to the retrospective design, detailed information on distal rectal polyp burden could not be retrieved, and it was therefore not possible to determine its impact on surgical decision-making. Nevertheless, as there was no significant difference in colon polyp density between the groups, we concluded that the choice of anastomosis type was more likely determined by surgeon preference rather than distal rectal polyp severity.

Our findings suggest that stapled IPAA, despite a higher risk of adenoma recurrence at the anal transitional zone, provides superior functional outcomes and shorter operative time compared to hand-sewn IPAA. Importantly, all recurrent adenomas in the stapled group were successfully managed without progression to carcinoma under regular endoscopic surveillance, underscoring the critical role of surveillance programs. A particular strength of our study is that both oncological and functional outcomes were assessed simultaneously, with oncological safety tracked over a lifelong follow-up period. Therefore, in centers where long-term endoscopic follow-up can be reliably implemented, stapled IPAA may be considered the preferred surgical option for FAP patients.

## Conclusions

In conclusion, all recurrent adenomas in the stapled group were successfully managed without progression to carcinoma although adenoma recurrence at the anal transitional zone occurred more frequently in the stapled group compared with the hand-sewn group. In addition, stapled IPAA was associated with shorter operative time and better functional outcomes regarding fecal incontinence. Taken together, these results suggest that stapled IPAA may be regarded as a reasonable surgical option for FAP patients when combined with regular endoscopic surveillance.

## Data Availability

No datasets were generated or analysed during the current study.
